# Image-Based Dietary Assessment Using the Swedish Plate Model: Evaluation of Deep Learning–Based You Only Look Once (YOLO) Models

**DOI:** 10.2196/70124

**Published:** 2025-08-14

**Authors:** Gustav Chrintz-Gath, Meena Daivadanam, Laran Matta, Steve McKeever

**Affiliations:** 1 Department of Informatics and Media, Uppsala University, Box 513, Uppsala, 75120, Sweden, 46 709901474; 2 Global Health and Migration Unit, Department of Women’s and Children’s Health, Uppsala University, Uppsala, Sweden

**Keywords:** food recognition, object detection models, dietary assessment, You Only Look Once, deep learning, machine learning

## Abstract

**Background:**

Recent advances in computer vision, particularly in deep learning, have significantly enhanced object recognition capabilities in images. Among these, real-time object detection frameworks such as You Only Look Once (YOLO) have shown promise across various domains. This study explores the application of YOLO-based object detection for food identification and portion estimation, with a focus on its alignment with the Swedish plate model recommended by the National Food Agency.

**Objective:**

The primary aim of this study is to evaluate and compare the performance of 3 YOLO variants (YOLOv7, YOLOv8, and YOLOv9) in detecting individual food components and estimating their relative proportions within images, based on public health dietary guidelines.

**Methods:**

A custom dataset comprising 3707 annotated food images spanning 42 food classes was developed for this study. A series of preprocessing and data augmentation techniques were applied to enhance dataset quality and improve model generalization. The models were evaluated using standard metrics, including precision, recall, mean average precision, and *F*_1_-score.

**Results:**

Among the evaluated models, YOLOv8 outperformed YOLOv7 and YOLOv9 in both peak precision and *F*_1_-scores. It achieved a peak precision of 82.4%, compared with 73.34% for YOLOv7 and 80.11% for YOLOv9, indicating superior accuracy in both food classification and portion estimation tasks. YOLOv8 also demonstrated higher confidence in its predictions. However, all models faced challenges in distinguishing visually similar food items, underscoring the complexity of fine-grained food recognition.

**Conclusions:**

While YOLO-based models, particularly YOLOv8, show strong potential for food and portion recognition aligned with dietary models, further refinement is needed. Improvements in model architecture and greater diversity in training data are essential before these systems can be reliably deployed in health and dietary monitoring applications.

## Introduction

The rise in type 2 diabetes presents a significant public health concern both globally and in Sweden, the context of this research [1]. Socioeconomically disadvantaged suburbs are disproportionately affected; these areas tend to be multicultural and have a high proportion of immigrants. Despite efforts to promote healthy eating behaviors, many individuals struggle to make informed dietary choices due to a range of individual socioeconomic barriers, as well as broader food environment factors. Additionally, dietary assessment approaches often rely on subjective self-reporting methods that carry a high respondent burden, leading to inaccuracies and inconsistencies in data collection and analysis [2]. This poses a challenge to accurately understanding and addressing the dietary needs and behaviors of diverse populations, thereby impeding the development of targeted interventions and policies to combat the escalating rates of type 2 diabetes and related health issues [3].

We aim to address the lack of effective tools and methodologies for accurately identifying food components within images to assess dietary patterns among multicultural populations. This gap highlights the need for innovative approaches that leverage advanced technologies, such as machine learning and computer vision, to develop robust solutions for dietary assessment and intervention. Object detection in food images involves applying these advanced technologies to identify and localize different food items within an image. The purpose of our work was to evaluate the performance of You Only Look Once (YOLO) models in predicting the adherence of food images to the Swedish plate model. The plate is widely used as a model to convey dietary guidelines and serves as a practical proxy for assessing the healthiness of a meal. By leveraging machine learning techniques, we aim to provide insights into the effectiveness of object detection algorithms in dietary assessment and contribute to the development of innovative solutions that promote healthy eating habits.

Traditionally, food intake has been measured using food diaries, food frequency questionnaires, and other tools that, while informative, place a high burden on respondents and are prone to inaccurate reporting. We seek a less obtrusive approach to dietary self-reporting—one that is automated and relies solely on photos of food to determine whether one’s diet is improving.

In recent years, there have been major breakthroughs in machine learning and computer vision, particularly in object detection and the development of deep learning techniques [4]. Object detection refers to the process of identifying and localizing objects within images or videos. This capability has diverse applications, ranging from surveillance and autonomous driving to medical imaging and dietary assessment.

Object detection has its roots in the early 1990s, when the first algorithms were developed. However, due to the limited quality of image representations at the time, no major models were constructed [5]. In 2001, the Viola-Jones Detector became the first real-time method for human face detection [6]. This was followed by the introduction of the histogram of oriented gradients detector [7], which was later superseded by the deformable part-based model [8], an extension of the histogram of oriented gradients detector. Since the 2010s, convolutional neural networks (CNNs) have formed the foundation for many successful object detection models. Among the most prominent are faster region-based CNN [9] and YOLO [10].

R-CNN is a 2-stage object detection system. In the first stage, it generates region proposals; in the second stage, it classifies these proposals and refines the bounding boxes. The regional proposal network is a component of the faster R-CNN architecture responsible for generating the region proposals. It takes the feature maps produced by the preceding convolutional layer of a CNN as input and outputs region proposals along with their objectness scores (ie, the probability that an object exists within a given region of the image). By contrast, YOLO is a real-time object detection system that is both fast and accurate. The main idea behind YOLO is to perform object detection on an image in a single forward pass through a CNN, rather than in multiple stages as done in faster R-CNN. While faster R-CNN is marginally more accurate than YOLO, it is also slower. More precisely, faster R-CNN performs better with small objects and instances involving significant overlap, owing to its more sophisticated region proposal and classification stages. Nonetheless, YOLO is highly efficient and capable of running at real-time speeds, even on low-end hardware. It is also easier to implement and has a relatively simple architecture compared with faster R-CNN. Over time, YOLO has evolved into a family of models that either supersede their predecessors or introduce new features such that the latest versions are competitive with faster R-CNN models [11]. Each member of the YOLO family is well supported, easy to install, and capable of detecting all the bounding boxes in a single pass. For these reasons, we chose to explore 3 recent YOLO models to evaluate which would be most suitable for food detection from images.

Existing work on dietary food intake assessment from images is extensive. Similar to our use case, NutriNet [12] and DeepFood [13] were deep learning–based models that offer practical solutions for automatically analyzing dietary intake from images captured using users’ smartphones. Their focus is on promoting healthier eating habits and supporting informed decision-making about dietary intake. A systematic review of deep learning for food image recognition and nutrition analysis in the context of chronic disease monitoring is provided in [14]. The review covers 57 selected articles, evaluated based on their approach, employed models, datasets, experiment procedures, and results. The overarching goal was to achieve multirecognition of food items. The survey draws 2 key conclusions: first, that 5 different model types were predominantly used (mask R-CNN, R-CNN, fast R-CNN, faster R-CNN, and YOLO); second, that the field is progressing rapidly.

One framework commonly used in dietary assessment is the Swedish plate model, known locally as “Tallriksmodellen.” It provides guidelines for constructing balanced meals based on the proportions of different food groups, with an emphasis on variety, portion sizes, and the relationship between food components [15]. The model divides the plate into different sections representing various food groups, promoting appropriate proportions for a well-rounded meal. Adhering to the Swedish plate model supports a balanced intake of essential nutrients, thereby promoting health and helping to prevent diet-related diseases. By leveraging advancements in computer vision, we aim to automate the analysis of food images to accurately identify food components within images and assess their adherence to the Swedish plate model.

This work was undertaken as part of the PREVENT project, which aims to cocreate, implement, and test an intervention targeting cardiometabolic diseases, such as type 2 diabetes, within the high-risk population of Region Uppsala. To evaluate changes in dietary behaviors in a “real-life” implementation trial, an innovative approach with low respondent burden was sought. The goal was to enable an assessment of broad dietary shifts over the intervention period by analyzing how the food components on participants’ food plates adhered to the Swedish plate model. Our approach holds potential to support public health initiatives aimed at promoting healthier dietary choices and preventing cardiometabolic diseases such as type 2 diabetes.

The primary objective of this study is to assess the performance of 3 YOLO models in accurately identifying food components within images and their proportions in relation to the Swedish plate model. This process involves 2 main stages: data collection and the evaluation of object detection algorithms. Data collection is a crucial step in developing and training machine learning models for object detection in food images, while evaluating the performance of object detection algorithms is essential for assessing their performance and comparing their effectiveness across different datasets and tasks.

The remainder of this paper is structured as follows. The “Methods” section introduces the YOLO framework for single-pass image classification and outlines the 3 versions used in our experiments. It also describes the data collection and analysis procedures. The “Results” section presents the data preprocessing steps, model configurations, and experimental results. Finally, the “Discussion” section discusses the broader implications of our findings in the context of the PREVENT project.

## Methods

### Overview

This section describes the methods used to evaluate real-time object detection models. We outline the 3 YOLO versions used, the creation of the custom dataset, and the approach taken to assess model performance.

### YOLO Model for Object Detection

At the core of the YOLO architecture lies a CNN that processes the entire image in a single pass, enabling simultaneous localization and classification of objects. This unified approach eliminates the need for complex region proposal networks and postprocessing steps, thereby streamlining the object detection pipeline and supporting real-time inference.

The YOLO architecture comprises several key components, each playing a crucial role in the detection process [16]. First, the convolutional layers are responsible for feature extraction, capturing hierarchical representations of the input image. These layers leverage convolutional filters to detect meaningful patterns and features, such as edges, textures, and shapes, which are essential for object recognition. By hierarchically stacking multiple convolutional layers, the network learns increasingly abstract and discriminative features, enabling it to distinguish between different object classes and background clutter effectively. Second, the downsampling layers play a pivotal role in reducing the spatial resolution of feature maps while increasing their receptive field size. This downsampling process enables the model to capture more spatial information at multiple scales, which enhances its ability to detect objects of varying sizes and aspect ratios. Additionally, down-sampling facilitates the integration of contextual information from surrounding regions, enabling more robust and accurate object localization. Finally, the detection layers are responsible for generating the bounding box coordinates and class probabilities for detected objects. These layers typically consist of a combination of convolutional and fully connected layers, followed by specialized activation functions such as sigmoid or softmax (a mathematical function used in machine learning algorithms to convert a vector of numbers into a probability distribution). The output from the detection layers comprises a set of bounding boxes, each associated with a confidence score and corresponding class probabilities.

An essential aspect of optimizing YOLO models is the tuning of hyperparameters, which are external settings that influence both the training process and the model architecture [17]. Key hyperparameters include learning rate, batch size, number of epochs, and YOLO-specific parameters such as backbone architecture and object confidence thresholds. Proper tuning of these parameters is crucial, as it directly impacts the model’s learning efficiency and overall performance. For example, an inappropriate learning rate may lead to suboptimal convergence, while unsuitable batch sizes can impact training stability. Hyperparameter optimization is therefore vital for achieving high performance in YOLO-based object detection tasks, ensuring the model functions both robustly and accurately.

Since its initial publication in 2016, successive versions of the YOLO architecture have been developed to further enhance performance. Each version features distinct architectural and network design elements, with their own characteristics and capabilities. Hence, when researchers have evaluated these models on specific datasets, newer versions have not always demonstrated superior performance [18].

YOLOv7 introduces modifications to the efficient layer aggregation network (ELAN) architecture [19,20], resulting in the extended efficient layer aggregation network (E-ELAN). Unlike ELAN, the E-ELAN architecture incorporates the principles of expand, shuffle, and merge cardinality to improve model learning while preserving gradient flow paths. In its computational blocks, E-ELAN uses group convolution to expand both channel dimensions and cardinality, applying a consistent channel multiplier and group parameter across all blocks in a layer. This strategy ensures scalability and enhances the model’s adaptability across diverse datasets.

The YOLOv8 model was developed by Ultralytics, the same team behind YOLOv5. YOLOv8 introduces modifications to the cross-stage partial (CSP) modules [21]. Its feature map module combines a simpler algorithm than that used in YOLOv5 with one adapted from YOLOv7. This strategic integration enhances model efficiency while maintaining strong performance. By leveraging the strengths of both architectures, YOLOv8 achieves a refined balance between computational complexity and feature representation. These adaptations improve training dynamics, scalability, object detection accuracy, and processing speed.

YOLOv9 builds on the foundation of YOLOv5 by introducing 2 key innovations: programmable gradient information (PGI) and the generalized efficient layer aggregation network (GELAN) [22]. These design choices allow users to select appropriate computational blocks depending on the target inference device [23]. Together, PGI and GELAN address the problem of information loss in deep neural networks, ensuring high efficiency, accuracy, and adaptability. PGI helps preserve essential data across network layers, while GELAN optimizes parameter utilization and computational efficiency.

### Data Collection

The selection, preparation, and preprocessing of the dataset play a pivotal role in ensuring the models’ effectiveness and generalizability [24]. The goal is to create a comprehensive and representative dataset that captures a diverse range of food items and dishes, thereby facilitating robust model training and evaluation. It was also essential to address inherent imbalances and variability within food classes.

The custom-created dataset used in this study was mainly sourced from publicly available repositories, including Roboflow Universe [25] and Kaggle [26]. It comprised 3707 images spanning 42 different food classes. These images were collected from a variety of settings and contexts—some depicting home-cooked meals, though the majority featured simpler food items. The dataset was then divided into training, validation, and test sets. The training set mainly comprised images from the public repositories.

Care was taken to include images that reflected a range of real-world conditions. These included variations in lighting, partial occlusions (eg, partially visible food items), diverse backgrounds, and inconsistent plate arrangements. Such real-world variations can impact object detection accuracy and the reliability of nutritional estimations. Nonetheless, this diversity helps simulate typical user-captured meal images and promotes better generalization by the detection models. However, we acknowledge that these variations were not formally quantified. Future work should thus incorporate a more systematic curation and analysis of environmental diversity and its effect on model performance.

The validation set consisted of anonymized images of complete meal plates, including a diverse range of multicultural foods, to better reflect real-world model usage. These images were collected through private networks by 2 bachelor students conducting thesis work within the PREVENT project. The annotation process involved manually labeling each food item in the images to create ground truth data for training and evaluation. This included drawing bounding boxes around individual items and assigning them the appropriate class labels. Annotation was performed using the Roboflow tool [27], which enabled annotation on the entire dataset and export into COCO JSON format. The resulting annotations captured essential information about the type and quantity of food items present in the images, allowing the models to learn and recognize food components.

The dataset was split into 3570 images for training, 131 for validation, and 6 for testing, covering a broad spectrum of dietary items. The test set comprised just a few images from the PREVENT group, used primarily for inference testing, allowing the model to be evaluated on individual images. Additionally, the dataset included annotation labels corresponding to the location, name, and size of food items within the images, facilitating supervised learning.

Preprocessing techniques have a noticeable impact on dataset quality, especially given the presence of noise in images and the influence of uncontrolled/unlearned environmental factors [28]. The preprocessing steps applied were *Auto-Orient*, *Resizing*, and *Auto-Adjust Contrast*. *Auto-Orient* removes EXIF-based rotations and standardizes pixel ordering. This is important, as each image contains metadata that dictate the orientation in which it is displayed relative to how the pixels are arranged on disk [29]. *Auto-Orient* eliminates any misalignment between the image and its annotations. Next, *Resizing* downsizes images to smaller, standardized dimensions, resulting in faster training. Finally, *Auto-Adjust Contrast* boosts contrast based on the image’s histogram to improve normalization and line detection in varying lighting conditions. This is mainly used to enhance edge detection.

Data augmentation was used to create new training examples for the models to learn from by generating augmented versions of each image in the training set. The techniques used were *Rotation*, *Grayscale*, and *Noise*. *Rotation* introduced image variations rotated by 15°, helping the model detect objects even when images are not perfectly aligned [30]. *Grayscale* converted the image color to grayscale in 10% of the cases, adding variance to the training set. Lastly, *Noise* added random distortions to the images, helping the model become more resilient to imperfections that are typically ignored by the human eye.

### Analysis

The evaluation and analysis of the YOLO models’ performance in identifying food components and predicting their proportions based on the Swedish plate model is vital for assessing our methodology. The Pascal Visual Object Classes Challenge [31] has played a pivotal role in defining standard evaluation protocols and metrics for object detection tasks. It aims to provide a benchmark dataset for assessing object detection algorithms through standardized evaluation metrics. The selected evaluation metrics are precision, recall, mean average precision (mAP), and *F*_1_-score. Together, these offered a comprehensive insight into the models’ ability to detect and classify food components, an essential step for estimating proportions according to the Swedish plate model.

*Precision* is a metric that measures the accuracy of the model’s positive predictions. It is crucial for ensuring that the model accurately identifies and classifies food items without generating an excessive number of false positives. Precision is calculated as the ratio of true-positive predictions to the total number of positive predictions, indicating the proportion of correctly identified instances among all instances predicted as positive [32]. A high precision value indicates that the model makes fewer incorrect predictions, resulting in more reliable and trustworthy outcomes [33]. By evaluating precision, we can assess the model’s ability to make accurate and precise predictions regarding the presence of food items in images.


$$\text{Precision}=\frac{\text{TruePostive}}{\text{TruePostive}\;+\;\text{FalsePostive}}$$

*Recall* measures the model’s ability to correctly identify all relevant instances of a particular class [32]. It is calculated as the ratio of true-positive predictions to the total number of actual positive instances in the dataset, indicating the completeness of the model’s predictions:


$$\text{Recall}=\frac{\text{TruePostive}}{\text{TruePostive}\;+\;\text{FalsePostive}}$$

In the context of food image analysis, recall is essential to ensure that the model captures all food items present in the images, minimizing the risk of overlooking important dietary components. A high recall value indicates that the model effectively detects and identifies most food items, thereby improving its usefulness for dietary assessment. By evaluating recall, we can assess the model’s ability to comprehensively detect and classify food items, contributing to a more holistic understanding of its predictive performance.

*The mAP* metric is widely used for evaluating the performance of object detection models, particularly in scenarios involving multiple object classes and varying levels of difficulty [33]. It calculates the average precision for each class and then computes the mean of these values, providing a comprehensive measure of the models’ overall performance.

$\text{Average Precision (AP)}=\frac{1}{N}\sum_{k=1}^{N}P(k)$


$$\text{mean}\;\text{Average}\;\text{Precision}\;(mAP)=\frac{1}{Q}\sum_{q=1}^{Q}AP(q)$$

where *N* is the number of relevant items; *Q* is the number of classes; *P* is the precision; and *K* is the relevant item.

There exist multiple variants of the *mAP* metric depending on the intersection over union (IoU) thresholds. The two used in this study are *mAP@*[0.5] and *mAP@*[0.5:0.95]. *mAP*@[0.5] is calculated at a fixed IoU threshold of 0.5, and measures the model’s ability to accurately localize objects. It evaluates the overlap between predicted bounding boxes and ground truth boxes, requiring at least 50% overlap for a correct detection. By contrast, *mAP*@[0.5:0.95] is computed over varying IoU thresholds, ranging from 50% to 95%. This provides insights into the model’s performance under varying levels of localization strictness in bounding box overlap criteria, and offers a more comprehensive view of detection performance across different difficulty levels [32]. The mean average precision was chosen because of its ability to consider the average precision across all classes while providing a holistic measure of the models’ effectiveness and robustness in food image analysis, facilitating informed decision-making and dietary recommendations.

The *F*_1_-score is a commonly used metric in binary classification tasks, including object detection. It provides a balance between precision and recall by considering both false positives and false negatives in the model’s predictions. The *F*_1_-score is particularly valuable when class distribution is imbalanced or when there is a trade-off between precision and recall [32]. In this study, the *F*_1_-score was used to evaluate the overall accuracy and effectiveness of the YOLO models in detecting and classifying food items within images. By combining both precision and recall into a single metric, the *F*_1_-score offers a more comprehensive assessment of the models’ predictive performance, enabling a more nuanced evaluation of their predictive capabilities.


$$\text{F1}\;\text{score}=2\times \frac{\text{Precision}\times \text{Recall}}{\text{Precision}+\text{Recall}}$$

### Ethical Considerations

This study involved the development and evaluation of deep learning models for dietary assessment using a custom image dataset. The dataset comprises images of food items that do not contain any identifiable personal information, human participants, or metadata linked to individuals. All images were either sourced from publicly available repositories or captured in controlled settings without the inclusion of human participants. Therefore, ethical approval and informed consent were not required, in accordance with the guidelines of the Swedish Ethical Review Authority (Etikprovningsmyndigheten), available online [34].

## Results

### Overview

This section presents the results of our study. We begin by outlining the model configurations, including architectural choices and hyperparameter settings. This is followed by a summary of the experiments and a detailed analysis of the performance metrics for each model.

### Model Configuration

In addition to extensive data augmentation techniques, such as rotation, noise injection, and grayscale conversion, we applied transfer learning by initializing the YOLO models with weights pretrained on the COCO dataset. This large-scale image recognition dataset was designed for various tasks, including object detection, and enhances generalization for the food detection task. Training was performed using an NVIDIA 3070 GPU with 16 GB VRAM, which offers significant computational advantages by speeding up model convergence and reducing training time. Hyperparameters define key aspects of the learning process. The backbone, as described in the “Methods” section, is a critical architectural component of each YOLO model. It directly influences the model’s ability to capture intricate features and patterns in the input data, thereby affecting overall detection accuracy and robustness [18]. Training epochs, which denote the number of complete passes through the dataset during training, were set to 50 for all 3 models. The batch size, that is, the number of samples processed before the internal model’s parameters are updated, was set to 8 for both YOLOv7 and YOLOv9, and 16 for YOLOv8. The higher batch size for YOLOv8 was due to its more efficient memory utilization during training. Unlike YOLOv7’s E-ELAN and YOLOv9’s GELAN/PGI modules, YOLOv8 employs streamlined CSP modules, making it less computationally demanding per batch. This architectural efficiency allowed for a larger batch size without exceeding GPU memory limits. A larger batch size also helps stabilize gradient updates and can accelerate convergence, as observed during early training trials. Both epochs and batch size play a crucial role in determining the convergence behavior of the model during the training phase. An optimal configuration ensures sufficient iterations for learning while avoiding issues such as underfitting or excessive computational overhead. Similarly, adjusting the learning rate, weight decay, and momentum was essential for fine-tuning the model’s optimization process; these parameters were configured identically across all 3 models. Finally, the choice of image size had significant implications for both computational efficiency and detection performance, and was set to 640 for all 3 models.

### Performance Results

The 3 YOLO models were trained using a custom dataset consisting of images annotated with bounding boxes corresponding to various food items. The training process involved optimizing the model’s parameters to minimize a composite loss function, which included components for bounding box regression and object classification. Table 1 presents the metrics on a sample of the data before training, while Table 2 shows the same metrics after training was completed, highlighting the superior performance of YOLOv8.

**Table 1.** Lowest results at the start of training.

Model	Precision	Recall	mAP^a^@[0.5]	mAP@[0.5:95]
YOLO^b^v7	0.02079	0.4463	0.04819	0.02756
YOLOv8	0.35552	0.40618	0.3243	0.22303
YOLOv9	0.40253	0.35802	0.35684	0.22947

a mAP: mean average precision.

b YOLO: You Only Look Once.

**Table 2.** Highest results at the end of training.

Model	Precision	Recall	mAP^a^@[0.5]	mAP@[0.5:95]	*F*_1_-score
YOLO^b^v7	0.7334	0.6863	0.6941	0.5446	0.65@0.419
YOLOv8	0.82382	0.67553	0.70447	0.53606	0.63@0.723
YOLOv9	0.80114	0.64477	0.6892	0.55459	0.64@0.480

a mAP: mean average precision.

b YOLO: You Only Look Once.

### Detailed Performance Metrics

#### Performance Metrics Across Model Variants

**Figure 1.**
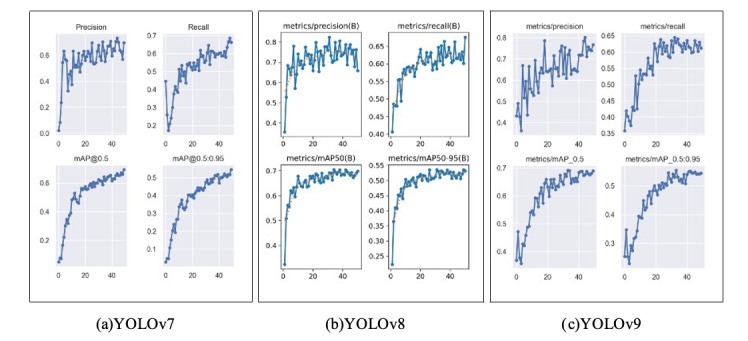
Performance metrics overview. The x-axis represents the number of epochs, and the y-axis represents the metric value. mAP: mean average precision; YOLO: You Only Look Once.

For each of the 3 models, the detailed progression of performance metrics is shown in Figure 1A-C. The validation set consists of real-world food dishes, making the resulting metrics meaningful, as they reflect the models’ ability to perform in practical, everyday scenarios.

#### Precision

Precision measures the accuracy of the model’s positive class predictions. For YOLOv7, it started very low at 0.02079, as expected during the early stages of training, but gradually improved over the epochs (iterations), peaking at 0.7334 by epoch 44. This indicates that 73.34% of the model’s positive predictions were correct, reflecting its ability to accurately identify relevant objects in the dataset.

For YOLOv8, precision started low at 0.35552 and gradually improved over the epochs. It peaked around the midpoint of training, reaching 0.82382 at epoch 31, while the final epoch showed a precision of 0.6584. This indicates that at its best, 82.382% of the model’s positive predictions were correct.

YOLOv9 started with a higher precision of 0.43127. Interestingly, this was not its lowest value—that occurred at epoch 3, where it dropped to 0.40253. Precision then steadily increased, peaking at 0.80114 in epoch 45, and ending at 0.76679 in the final epoch. This indicates that at its best, 80.114% of the model’s positive predictions were correct.

#### Recall

Recall evaluates the model’s ability to capture all relevant instances. For YOLOv7, it started at 0.4463 and experienced fluctuations before stabilizing and reaching its highest value of 0.6863 by epoch 48. This indicates that the model correctly identified around 68.63% of all positive instances in the dataset.

YOLOv8 started with a recall of 0.40618 and steadily improved, reaching its highest value of 0.67553 at epoch 50 (the final epoch). This indicates that the model correctly identified around 67.553% of all positive instances in the dataset.

Meanwhile, YOLOv9 began with its lowest recall value of 0.35802 and then steadily increased, peaking at 0.64477 at epoch 33. By the final epoch, it reached 0.61206, indicating that the model correctly identified around 61.206% of all positive instances.

#### Mean Average Precision

The mAP provides insights into the model’s overall detection performance. At an IoU threshold of 50%, YOLOv7’s mAP steadily increased from 0.04819 to 0.6941 by epoch 50, indicating consistent improvement in object detection accuracy. Similarly, the mAP for IoU thresholds ranging from 0.5 to 0.95 rose from 0.02756 to 0.5446 over the training epochs, reflecting the model’s ability to maintain precision across a broader range of IoU criteria.

At an IoU threshold of 50%, YOLOv8’s mAP steadily increased from 0.3243 in the first epoch, reaching its peak of 0.70447 by epoch 38. Similar to the precision value, it peaked slightly past the midpoint of training and ended at 0.69816 by epoch 50. Similarly, the mAP at IoU thresholds ranging from 0.5 to 0.95 rose from 0.22303 in the first epoch, peaked at 0.53606 in epoch 31, and settled at 0.53174 in the final epoch, demonstrating the model’s ability to maintain strong detection performance across varying IoU levels.

For YOLOv9, the mAP at an IoU threshold of 50% increased steadily throughout training, reaching its highest value of 0.6892 in the final epoch. For the mAP at IoU thresholds ranging from 0.5 to 0.95, performance improved consistently until peaking at 0.55459 in epoch 34, before ending at 0.54468 by epoch 50. These results reflect the model’s ability to maintain precision across varying levels of detection difficulty.

#### *F*_1_-Score

**Figure 2.**
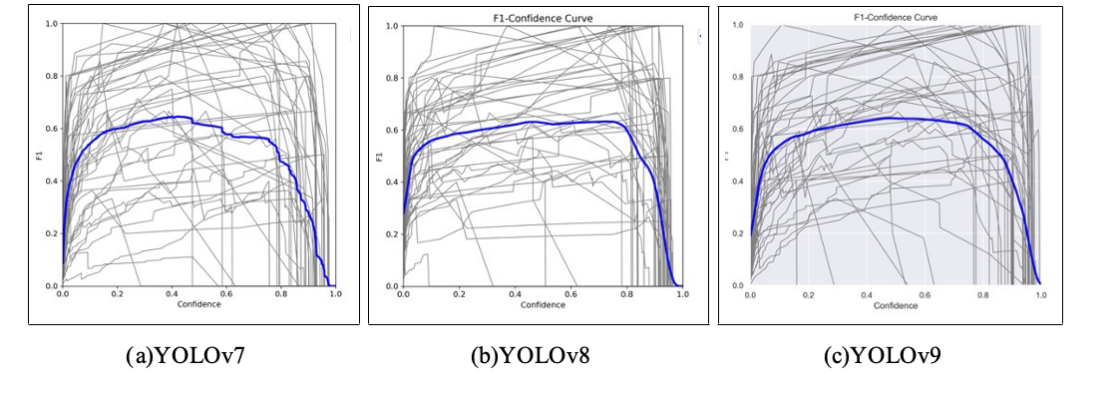
*F*_1_-scores. The x-axis represents the confidence score, and the y-axis represents the *F*_1_-score. YOLO: You Only Look Once.

The *F*_1_-scores, shown in Figure 2A-C, reflect the harmonic mean of precision and recall. For YOLOv7, the *F*_1_-score peaked at 0.65 at a confidence level of 0.419. This suggests a balanced performance between precision and recall, demonstrating the YOLOv7 model’s effectiveness in both identifying relevant objects and minimizing false positives. For YOLOv8, the *F*_1_-score reached its peak of 0.63 at a confidence level of 0.723, indicating a slightly better performance between precision and recall at higher confidence thresholds. Finally, the *F*_1_-score for YOLOv9 reached its peak of 0.64 at a confidence level of 0.480, similar to YOLOv7, although the curves appear quite different. The tailing-off pattern observed in Figure 2A for YOLOv7’s *F*_1_-score may be indicative of overfitting during the final epochs of training. Unlike YOLOv8 and YOLOv9, which maintain more stable *F*_1_-scores, YOLOv7 exhibits a decline after its peak, suggesting that the model may have begun to memorize training patterns rather than generalize effectively. This behavior could also be attributed to its architectural configuration, where the extended E-ELAN modules may have increased model complexity relative to the training data size, making it more prone to such fluctuations.

#### Confusion Matrix

**Figure 3.**
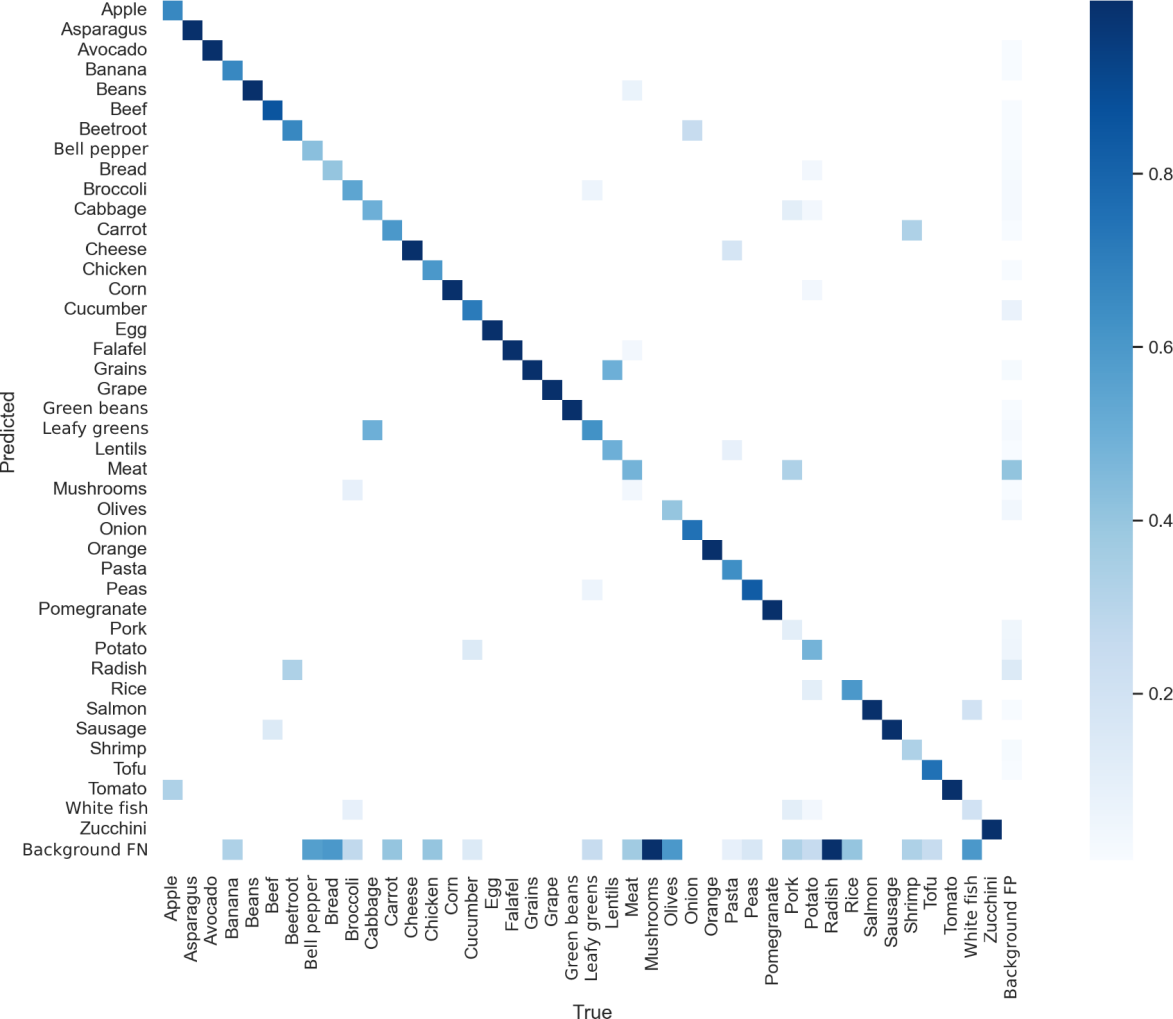
YOLOv7 confusion matrix. Darker colors indicate a higher number of correct predictions by the food plate algorithm. Squares off the diagonal represent misclassifications. Background false negative (FN) indicates that the model fails to detect a true object that is present in the image, leaving it as part of the background. Background false positive (FP) indicates that the model incorrectly predicts an object when there is none (ie, it finds an object in the background). YOLO: You Only Look Once.

A confusion matrix visually represents the model’s performance across different classes using the validation set. Each box represents the model’s prediction for a specific class, with predicted classes displayed along the y-axis and actual classes along the x-axis. A strong diagonal trend reflects accurate predictions, where the predicted class matches the true class. The darker the color, the greater the number of times that class was predicted, relative to the actual frequency of that class in the dataset. This visualization offers insights into the model’s strengths and weaknesses in classifying different objects. For YOLOv7, as shown in Figure 3, most classes are accurately predicted according to their true labels. However, there are classes—such as mushroom and radish—where the model misclassified them as background. These misclassifications may stem from a combination of factors. One plausible reason is the one-object-per-grid-cell constraint inherent in YOLO architectures, which may lead to the suppression of smaller or overlapping objects, particularly when they are clustered with more dominant items on the plate. Additionally, the underrepresentation of these classes in the training dataset likely contributed to reduced recognition accuracy. When certain food categories appear infrequently, the model struggles to learn their distinct visual features, increasing the likelihood of ignoring them entirely during prediction.

**Figure 4.**
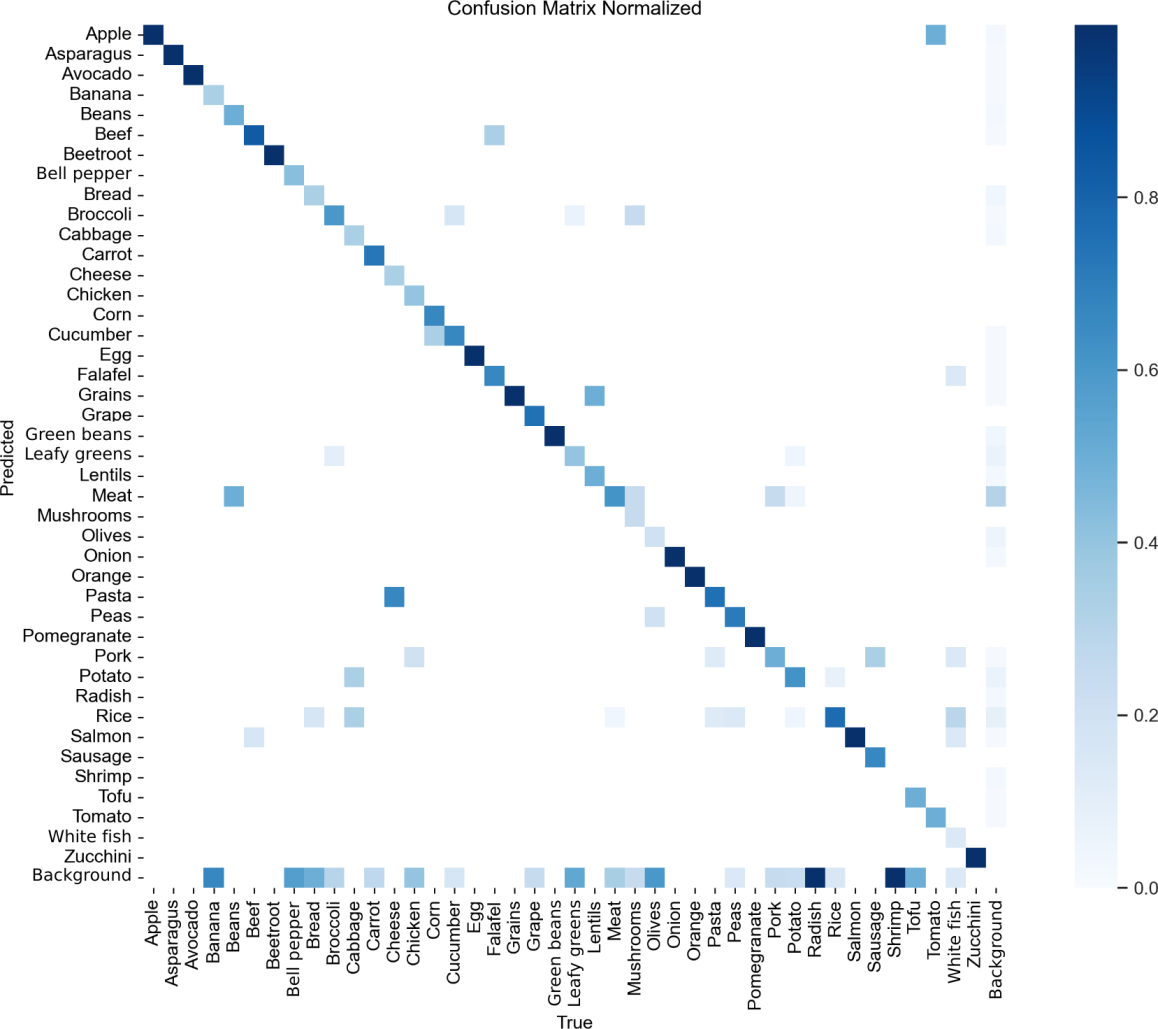
YOLOv8 confusion matrix showing that most classes are accurately predicted. YOLO: You Only Look Once.

For YOLOv8, shown in Figure 4, most classes are accurately predicted based on their true labels. However, the predictions show slightly more dispersion compared with YOLOv7’s confusion matrix. Notably, classes such as radish and shrimp were occasionally predicted as background. Such misclassifications typically occur when the model fails to detect a component that is actually present in the image.

**Figure 5.**
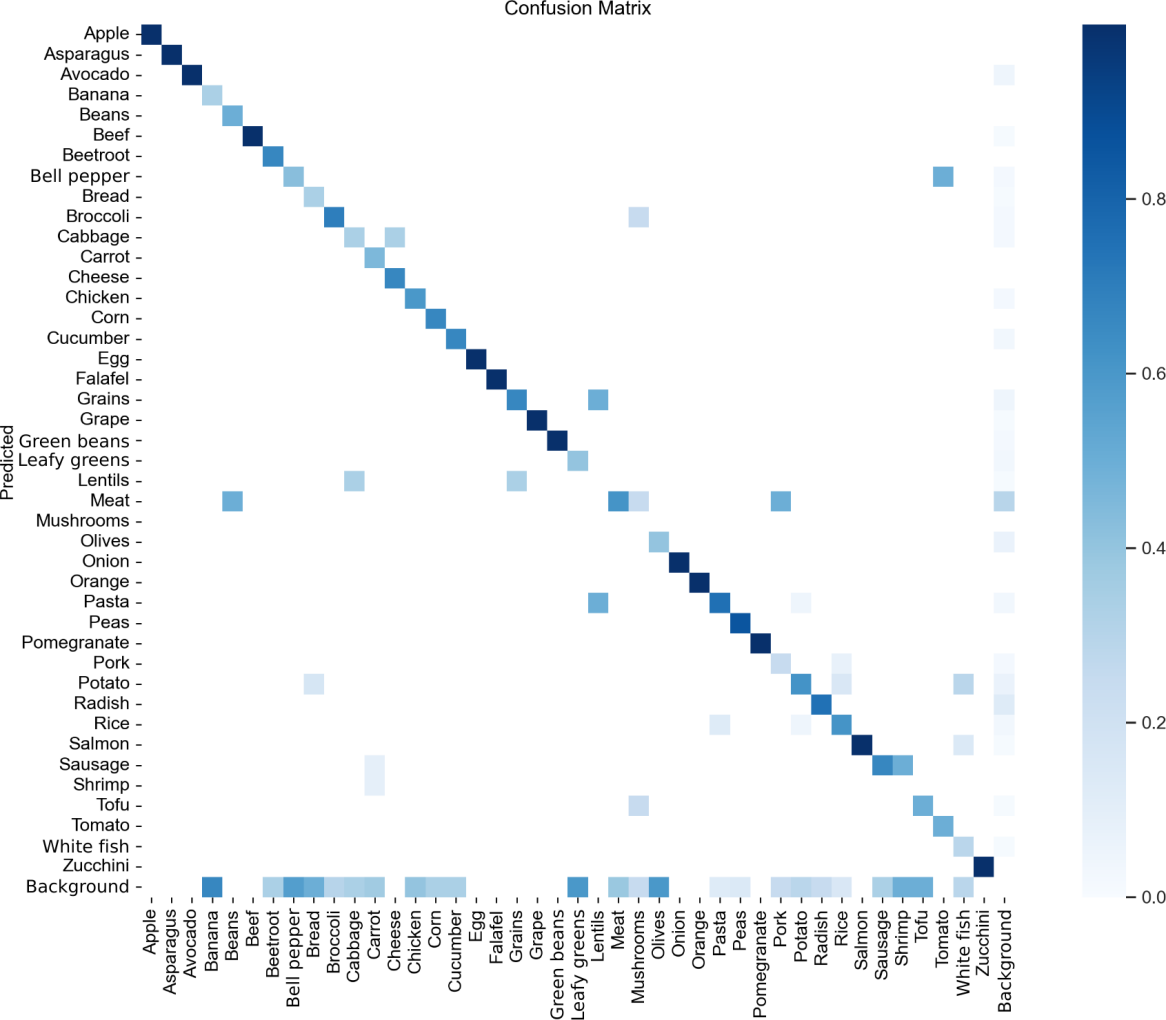
YOLOv9 confusion matrix demonstrating good predictability, but less than YOLOv7 and YOLOv8. YOLO: You Only Look Once.

Figure 5 shows the matrix for YOLOv9. Most classes are accurately predicted based on their labels, although there are more incorrect predictions than for either YOLOv7 or YOLOv8, where the model missed components in the image and therefore categorized them as background. There are no distinct classes where the model missed all of the instances, such as in the previous models, where both had 2 classes each that were wrong.

### Summary of YOLO Models

Based on the results from the performance metrics—precision, recall, mAP, and *F*_1_-score—all 3 YOLO models (YOLOv7, YOLOv8, and YOLOv9) demonstrated competitive performance in identifying and classifying food components. However, YOLOv8 emerges as the most suitable model overall. With a peak precision of 82.382%, YOLOv8 exhibits high accuracy in identifying positive instances, which is essential for classifying food components within images and estimating their proportions according to the Swedish plate model. While YOLOv7 exhibits the highest recall rate, YOLOv8 closely follows, indicating its effectiveness in capturing relevant objects. Additionally, YOLOv8 surpasses YOLOv7 in mAP, achieving a peak value of 0.70447 at the 50% IoU threshold, highlighting its capability to localize objects with high confidence. Although its *F*_1_-score is slightly lower compared with those of YOLOv7 and YOLOv9, YOLOv8’s higher optimal confidence level (0.723) suggests stronger certainty in its predictions, an advantage in practical applications that require reliable food classification.

As the models were trained using similar hyperparameters and the same dataset, the observed performance differences can be attributed primarily to architectural innovations. YOLOv8 employs a modified CSPDarknet backbone, which enhances feature representation while maintaining computational efficiency. This architecture supports improved gradient flow and better preservation of spatial information, both critical for distinguishing visually similar food items, such as lentils and grains. By contrast, YOLOv7’s E-ELAN and YOLOv9’s GELAN/PGI, although powerful, may introduce added complexity or optimization challenges that slightly hinder detection precision in this domain. YOLOv8’s combination of a lightweight yet expressive architecture and more stable learning dynamics likely contributes to its superior performance in food detection tasks, which demand both fine-grained object recognition and real-time speed. Future ablation studies could help isolate the contributions of specific architectural elements, such as CSP modules and feature pyramid designs, to better understand their roles in this performance gain.

## Discussion

### Principal Findings

This study evaluated the application of 3 YOLO-based object detection models for automated food recognition and portion estimation aligned with the Swedish plate model. Among the models, YOLOv8 demonstrated superior performance across several key evaluation metrics, achieving the highest precision and exhibiting greater confidence in its predictions compared with YOLOv7 and YOLOv9. These findings underscore the potential of modern deep learning architectures to accurately identify food items and estimate their proportions in plated meals, highlighting their promise for automated dietary assessment.

Dietary assessment plays a crucial role in public health, particularly in addressing the growing prevalence of type 2 diabetes [35]. In Sweden, as in many other countries, the incidence of type 2 diabetes and other cardiometabolic diseases has risen markedly, prompting efforts to develop effective strategies for prevention and management. Supporting individuals in adopting healthier behaviors and promoting active lifestyles is essential to curbing this trend. Enabling people at risk to easily monitor their food intake, such as through photo-based analysis, is an appealing option. This study evaluated the effectiveness of YOLO models in identifying food components within images and estimating their proportions according to the Swedish plate model, which offers a simple yet practical framework for encouraging balanced and healthy eating habits. The model divides the plate into sections representing various food groups, emphasizing appropriate portion sizes and proportions to support a well-balanced meal. By following this model, individuals can achieve a nutrient-rich diet that promotes overall health and helps prevent diet-related conditions. The performance metrics used in this study offered valuable insights into each model’s strengths and limitations, enabling a clear comparison of their ability to identify food components. Among the 3, the YOLOv8 model demonstrated the best overall performance and is therefore recommended for implementation in PREVENT’s pilot program.

### Comparisons With Prior Work

Our research aimed to investigate the potential of using object detection models to identify food components within images and estimate their proportions according to the Swedish plate model. This approach distinguishes our work from earlier contributions [12,13], as we trained our models specifically on this healthy meal planning template rather than a general food recognition dataset.

### Limitations

One limitation of our study was the restricted size of the food plate library folder, which resulted in many food items being underrepresented. This gap is reflected in the confusion matrices, which reveal uncertainties in food identification, indicating insufficient training data. Some foods, such as oranges, onions, and grapes, were identified with high accuracy. By contrast, lentils were often misclassified as pasta or grains, possibly because these categories were better represented in the training data or because their visual features are more distinct, leading the food plate algorithm to favor them. Another challenge was the identification of foods with similar appearance. For instance, red paprika was frequently misidentified as tomato, suggesting the absence of a dedicated class for (red) paprika. These findings highlight the need for expanding and refining the dataset to ensure the models are robust enough for accurate dietary data collection [36].

Another important limitation concerns the model’s performance under varying real-world conditions, such as different lighting scenarios, partial occlusions, and camera angles. While the validation set includes some images taken in uncontrolled environments, such as home kitchens and multicultural meal settings, these conditions were not systematically varied, tested, or curated.

It is also crucial to clarify that an object detection model does not directly predict the healthiness of food based on the Swedish plate model. Rather, by identifying food components and their bounding boxes, the model can estimate the relative area each food occupies. This information can then be used to analyze the proportions of different food groups on the plate. Based on this analysis, a healthiness score can be generated—for example, using a Likert scale from 1 to 10—allowing users to compare meals and track their dietary progress. However, assessing a plate is more nuanced than simply identifying its components and their respective proportions. Factors such as the content of salt or sugar, the type and amount of fat, and the cooking method also play important roles in determining a meal’s healthiness—yet these are difficult to evaluate through photographic analysis alone. Nevertheless, identifying the components of a meal from a photo can serve as a useful proxy for dietary assessment from a health perspective.

### Conclusions

The broader societal relevance of this study lies in its potential to enhance dietary assessment and health promotion initiatives. Applying machine learning techniques such as YOLO could enable more accurate and efficient methods for monitoring and improving dietary habits, thereby contributing to the prevention of diet-related diseases and the promotion of healthier lifestyles. By leveraging the principles of the Swedish plate model, this study extends the application of object detection toward the development of actionable and practical tools that support informed dietary choices. Future work should explore alternative object detection models beyond the YOLO series, such as faster R-CNN and cascade R-CNN [37]. Additionally, creating more comprehensive and diverse datasets is essential to improve the accuracy and robustness of these models, thereby expanding their potential utility.

## Abbreviations

CNN: convolutional neural network
CSP: cross-stage partial
E-ELAN: extended efficient layer aggregation network
ELAN: efficient layer aggregation network
GELAN: generalized efficient layer aggregation network
IoU: intersection over union
mAP: mean average precision
PGI: programmable gradient information
R-CNN: regions with CNN
YOLO: You Only Look Once
